# Unanticipated Residual Gastric Contents in an Appropriately Fasted Pediatric Patient on Glucagon-Like Peptide-1 Receptor Agonist Therapy: A Case Report and Review of Perioperative Considerations

**DOI:** 10.7759/cureus.99731

**Published:** 2025-12-20

**Authors:** Max Lookabaugh, Kim M Strupp, Leah Webb

**Affiliations:** 1 Anesthesiology, University of Colorado School of Medicine, Aurora, USA; 2 Pediatric Anesthesiology, University of Colorado Anschutz Medical Campus/Children's Hospital Colorado, Aurora, USA

**Keywords:** aspiration risk, delayed gastric emptying, gastric pocus, glp-1 receptor agonist, pediatric anesthesia, rapid sequence induction, semaglutide

## Abstract

Glucagon-like peptide-1 receptor agonists (GLP-1 RAs), including semaglutide (Wegovy®), are increasingly used for weight management in both adult and pediatric populations. Their mechanism of delayed gastric emptying raises concern for aspiration during anesthesia, yet current fasting guidelines provide limited direction for these patients. We present the case of a 14-year-old female patient with autism, developmental delay, and obesity managed with semaglutide who presented for esophagogastroduodenoscopy and colonoscopy. Despite holding semaglutide for 12 days, completing bowel preparation, and fasting from solids for 32 hours and clear liquids for 10 hours, preoperative gastric point-of-care ultrasound revealed a distended antrum containing fluid and particulate matter consistent with a full stomach. After multidisciplinary discussion and informed consent, the procedure proceeded with rapid sequence induction and endotracheal intubation to mitigate aspiration risk. Endoscopy confirmed substantial residual gastric contents exceeding 200 mL, though the procedure and anesthetic course were uneventful. This case underscores that standard fasting protocols may not ensure gastric emptying in patients on GLP-1 RA therapy, particularly during medication up-titration or in those with coexisting gastrointestinal motility disorders. Point-of-care gastric ultrasound proved valuable for individualized risk assessment and anesthetic planning. As the use of GLP-1 RAs expands, anesthesia teams should maintain heightened vigilance, consider extended fasting or gastric ultrasound when feasible, and engage in shared decision-making regarding perioperative management. Further research and updated guidelines are needed to define evidence-based fasting intervals and anesthetic strategies for this growing patient population.

## Introduction

Glucagon-like peptide-1 receptor agonists (GLP-1 RAs), such as semaglutide (Wegovy®), have quickly emerged as widely utilized pharmacologic agents in the management of both obesity and type 2 diabetes, including in pediatric populations [1,2]. Among their understood physiologic effects, delayed gastric emptying is especially relevant in the perioperative setting, as it may increase the risk of pulmonary aspiration during anesthesia. However, evidence describing the perioperative implications of GLP-1 RAs remains limited. 

Despite this possible risk, the current American Society of Anesthesiologists (ASA) fasting guidelines do not provide specific recommendations for patients on GLP-1 RAs [3]. Previous recommendations from the ASA in 2023 suggest pausing daily GLP-1 RAs on the day of surgery and pausing weekly formulations one week prior to elective procedures [4]. More recently, a 2024 multisociety clinical practice guidance advises that most patients can continue GLP-1 RAs before elective procedures; for some higher-risk scenarios, teams should consider a 24-hour liquid diet, gastric point-of-care ultrasound (PoCUS) when available, full-stomach precautions, or deferring elective care [5]. However, concrete data guiding appropriate fasting intervals, intraoperative management, and risk stratification in pediatric patients remain scarce. 

## Case presentation

We present the case of a 14-year-old female patient with autism spectrum disorder, developmental delay, attention-deficit/hyperactivity disorder, gastroesophageal reflux disease, binge eating, and obesity who presented for elective esophagogastroduodenoscopy (EGD) and colonoscopy to evaluate ongoing chronic abdominal pain and diarrhea. Her medical history included prediabetes with hemoglobin A1c (HbA1c) values ranging from 5.4% to 6.2% over the prior two years; however, she had no clinical diagnosis of diabetes and no symptoms of diabetic gastroparesis, making diabetes-related delayed gastric emptying unlikely. Although her history included abdominal pain and diarrhea, she had no documented symptoms suggestive of gastroparesis (such as chronic nausea, vomiting, early satiety, or postprandial fullness), making an underlying motility disorder less likely. Her home medications included escitalopram, amphetamine-dextroamphetamine, cetirizine, hydroxyzine, an oral contraceptive, magnesium oxide, riboflavin, and a multivitamin; none of these are associated with delayed gastric emptying, making semaglutide the only medication with known effects on gastric motility. 

**Figure 1:**
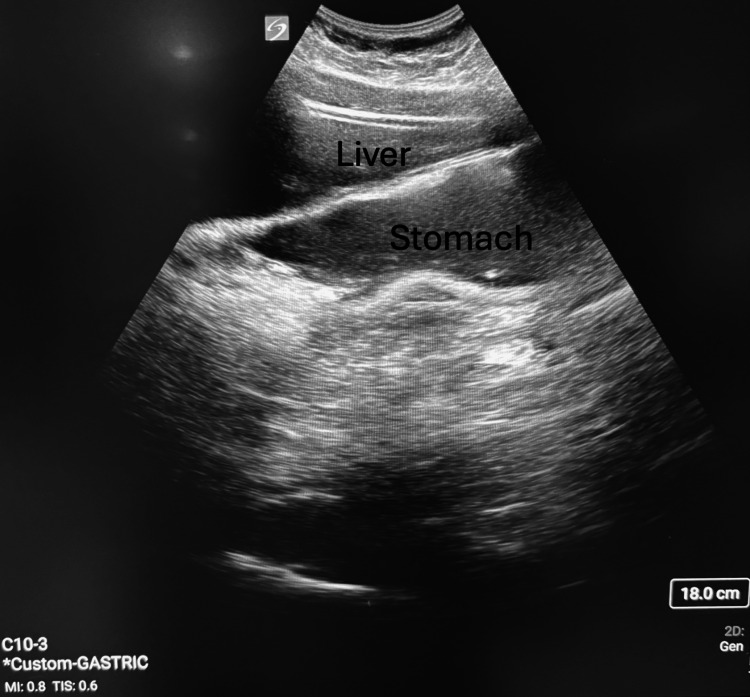
Preoperative gastric PoCUS demonstrating a distended antrum with mixed fluid and solid particulate matter. Stomach labeled with visible gastric antrum; note thick, hyperechoic layering consistent with particulate content. PoCUS: point-of-care ultrasound

The patient was undergoing treatment with semaglutide, initiated in April 2024 (three months prior to the procedure), for the treatment of obesity and binge eating. She was receiving a weekly formulation of semaglutide (1 mg once weekly). In addition to adhering to fasting guidelines and completing the bowel preparation protocol, the patient had held her semaglutide for 12 days. As a weekly formulation, her 12-day interruption represented more than one full dosing interval. The patient and mother reported adhering fully to the prep instructions, and there was no indication to believe that they had deviated from the plan. She followed the nil per os (NPO) guidelines and fasted from solids for 32 hours and clear liquids for 10 hours preoperatively, with a procedure start time of 16:00. Fasting times were confirmed through caregiver reporting, with the patient's caregiver providing direct supervision of all oral intake during the fasting period. Nonetheless, a preoperative gastric PoCUS exam was performed per the anesthesiologists' standard practice when caring for patients on GLP-1 RA medications. The antrum was found to be distended, in both supine and right lateral views, with a significant amount of gastric fluid with visible particulate matter, as shown in Figure 1. See Video 1 for a representative video demonstrating thick fluid and layering of particulate. Thus, the gastric antrum was classified as Grade 3 on the Perlas grading scale [6], indicating a full stomach with thick fluids or solids. The high risk of aspiration from the Grade 3 stomach contents merited discussion as to whether rescheduling the elective case would be beneficial. 

**Video 1:** Preoperative gastric PoCUS demonstrating a distended antrum with thick fluid and echogenic particulate, with visible layering during respiration. Duration: 5 seconds. No audio provided. All identifiers have been removed. View demonstrating thick, hyperechoic layering consistent with particulate gastric contents (Grade 3 stomach on the Perlas grading scale). PoCUS: point-of-care ultrasound

A comprehensive risk-benefit discussion was undertaken with the attending anesthesiologist, a second anesthesiologist with leadership in quality and patient safety and ASA gastric PoCUS course certification, the proceduralist, and the patient's guardian. Rescheduling the procedure, to allow for either longer medication washout or dose stabilization, would result in delaying the time-sensitive procedure for months. No additional opportunities for optimization were identified, as the patient had already followed the 2024 guidelines to adhere to a clear liquid diet [5], as well as the 2023 guidelines to hold the medication [4]. As such, further delay was deemed unlikely to result in significant risk reduction. The patient's guardian was informed of and accepted the higher risk of aspiration while agreeing that the benefit of proceeding likely outweighed the potential risks. 

**Figure 2:**
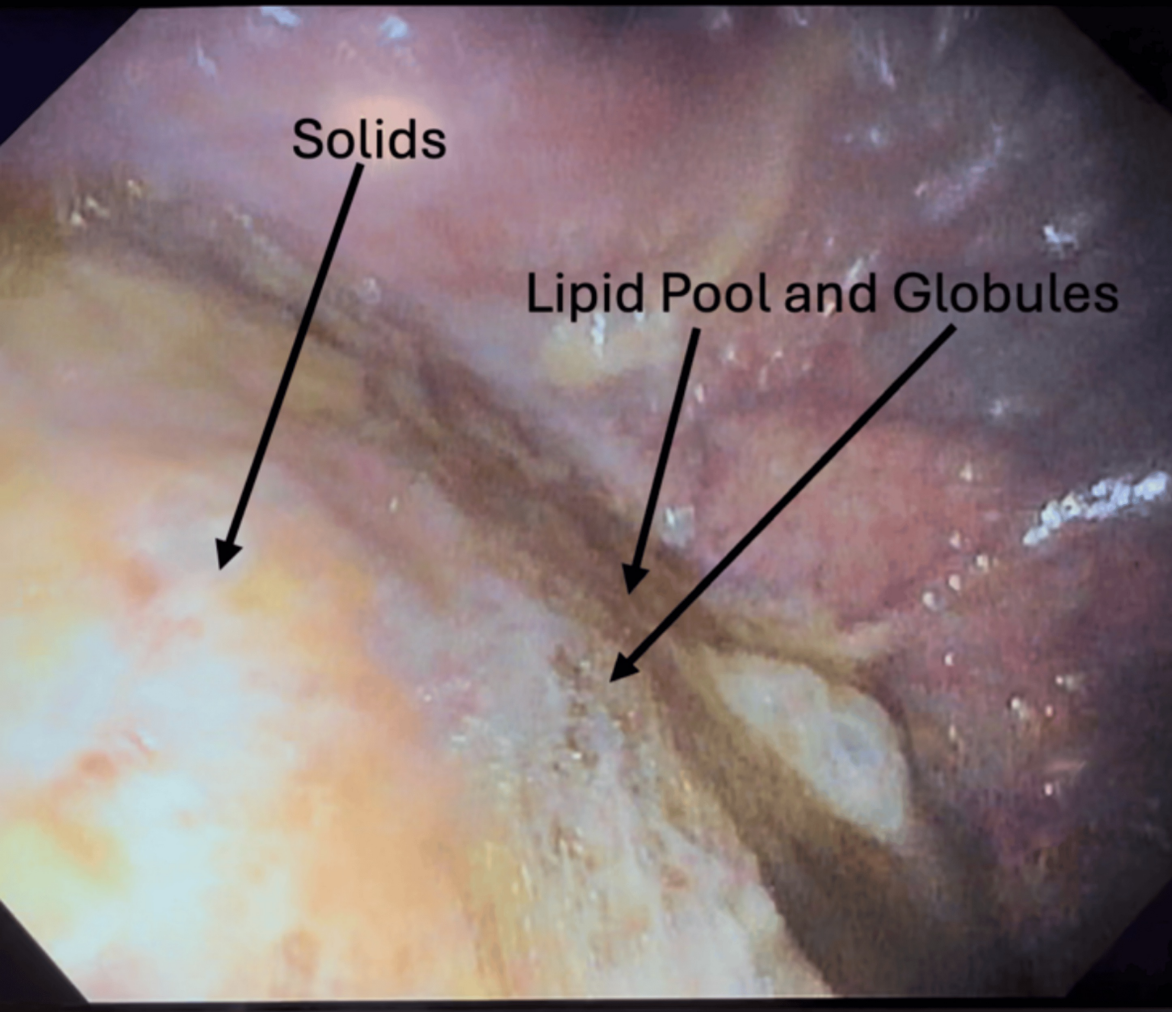
Endoscopic view showing retained gastric contents despite adherence to fasting and medication guidelines. Over 200 mL of thick, mixed gastric material suctioned; residual solids visible.

Thus, the decision was made to proceed with the planned procedures with a modification of the anesthetic plan. Typically, the anesthesiologist would use intravenous (IV) anesthesia with a natural airway for these procedures. However, to mitigate the aspiration risk, rapid sequence induction with endotracheal intubation was planned for the patient. After five minutes of preoperative oxygenation, and the placement of standard ASA monitors, IV induction was performed with the patient positioned with the head of the bed elevated to 45° and suction immediately available. A defasciculating dose of rocuronium and fentanyl was administered. Following that, propofol and a rapid push of succinylcholine were given. Following succinylcholine, tracheal intubation was achieved with a 6.5-mm cuffed endotracheal tube with no detectable aspiration of stomach contents. Subsequently, the endoscope was advanced from the esophagus through the gastroesophageal junction and into the stomach with mixed contents immediately visualized, as seen in Figure 2. More than 200 mL of gastric contents were suctioned from the stomach, with an inability to completely empty due to solid matter. The EGD and colonoscopy were otherwise completed without complications. The patient was extubated fully awake at the end of the procedure and transferred to the recovery room, where she was later discharged without sequelae. 

## Discussion

Our case highlights a growing challenge in the perioperative period: determining the ideal management of patients receiving GLP-1 RAs, who may have persistent delayed gastric emptying even after ostensibly appropriate medication withholding and fasting. In our patient, the failure of both standard bowel preparation and prolonged fasting to clear the stomach underscores how profound this delayed emptying can be in real-world practice. In the pediatric population, this risk may be compounded further by neurodevelopmental conditions or underlying gastrointestinal motility disorders, such as gastroparesis, which are more prevalent among children with neurologic and developmental disabilities [7]. Although our patient had no prior symptoms of chronic gastroparesis, we cannot entirely exclude baseline dysmotility; instead, GLP-1 RA therapy may have exacerbated an underlying motility vulnerability to produce the degree of gastric retention observed. Gastric PoCUS played a crucial role in providing timely, patient-specific information that informed the anesthetic planning and perioperative care of our patient. While not yet a routine part of perioperative evaluation, gastric PoCUS is gaining recognition as a valuable tool for assessing aspiration risk, especially in patients with non-standard risk profiles [8]. Despite adherence to fasting and medication hold recommendations, our pediatric patient exhibited a markedly distended stomach with particulate matter on preoperative gastric ultrasound. This underscores the uncertainty surrounding the appropriate perioperative management of patients receiving GLP-1 RAs. Although adult case reports have identified aspiration events associated with GLP-1 RA use [9,10], pediatric evidence remains sparse. 

Several recent studies reinforce the evolving understanding of this phenomenon. A 2025 Anesthesia & Analgesia analysis of preoperative gastric contents in GLP-1 RA users demonstrated increased residual volume compared with matched controls, despite guideline-compliant fasting [11]. Similarly, preliminary pediatric data presented at the 2025 Society for Pediatric Anesthesia meeting demonstrated that children on GLP-1 RAs showed markedly higher rates of thick fluid or solid contents on gastric PoCUS, even after guideline-compliant fasting [12]. These findings parallel those of our patient, in whom a Grade 3 antrum was observed even after >24 hours of fasting from solids and 12 days off medication. 

Adult case reports published by the Anesthesia Patient Safety Foundation further describe instances of semaglutide-associated gastric retention and regurgitation during anesthesia despite guideline-compliant fasting, underscoring the under-recognized aspiration risk of GLP-1 RAs [13]. Additionally, a 2025 Frontiers in Medicine retrospective analysis of more than 1,200 EGD cases found that GLP-1 RA users had significantly higher rates of retained gastric contents than controls, reinforcing the persistence of delayed gastric emptying despite appropriate fasting [14]. 

The 2024 Multi-society Clinical Practice Guidance on the perioperative management of GLP-1 RAs emphasizes a risk-stratified, multidisciplinary approach balancing metabolic benefit with aspiration risk [15]. The document recommends that care teams consider factors such as dose-escalation phase, high doses, gastrointestinal symptoms, or comorbidities that impair motility when deciding whether to continue or temporarily hold therapy. For patients deemed higher risk, the guidance advises a 24-hour liquid diet, assessment with gastric PoCUS when feasible, and the use of full-stomach precautions or rapid-sequence induction if proceeding with anesthesia. Complementing these recommendations, a 2024 review in the Journal of Clinical Medicine highlights the growing role of bedside gastric PoCUS to objectively evaluate aspiration risk and support individualized perioperative planning in both adult and pediatric populations [16]. Additional international guidance documents, including the 2025 Australian and New Zealand College of Anaesthetists (ANZCA) recommendations and the Society for Perioperative Assessment and Quality Improvement (SPAQI) multidisciplinary consensus statement, similarly advocate the individualized assessment and consideration of gastric ultrasound when managing patients on GLP-1-based therapies. 

Our case contributes to this growing body of evidence by highlighting that even with extended fasting and medication cessation, GLP-1-associated gastric retention can persist in children. Incorporating gastric PoCUS into preoperative evaluation allowed individualized risk stratification and informed decision-making regarding airway management and case timing. Further research is needed to determine whether longer medication holds, alternative dosing schedules, or real-time gastric assessment can meaningfully mitigate aspiration risk (Table 1). 

**Table 1:** Summary of the studies examining perioperative findings in GLP-1 RA users GLP-1 RA: glucagon-like peptide-1 receptor agonist; PoCUS: point-of-care ultrasound; RSI: rapid sequence induction; RGC: residual gastric content; EGD: esophagogastroduodenoscopy; NPO: nil per os

Ref.	Study/source	Design	Population/setting	Fasting/GLP-1 hold info	Assessment	Primary outcome	Key findings	Relevance to the present case
[7]	Corsello et al. (2023), Front Neurol	Narrative review	Children with neurologic impairment	Not GLP-1 specific; discusses delayed gastric emptying due to gut dysmotility	Literature synthesis	Evaluate nutritional and gastrointestinal management strategies in pediatric patients with neurologic disease	Identifies a high prevalence of delayed gastric emptying and feeding intolerance linked to autonomic and motility dysfunction	Highlights that neurodevelopmental comorbidities in our patient may exacerbate GLP-1-related gastric retention
[8]	Perlas et al. (2018), Can J Anaesth	Narrative review	General perioperative population	Not GLP-1 specific; reviews fasting guidelines and aspiration risk factors	Gastric PoCUS review	Summarize evidence for gastric ultrasound as a tool to assess aspiration risk	Supports gastric ultrasound as a reliable, noninvasive bedside technique for assessing residual gastric volume	Justifies the use of gastric PoCUS to guide anesthetic management and airway planning in our case
[9]	Sen et al. (2024), JAMA Surg	Cross-sectional observational	Adults on vs not on GLP-1 RAs, elective anesthesia	Standard fasting; some held up to 7 days	Gastric ultrasound	RGC	Higher RGC in GLP-1 users (~56% vs 19%); short holds not protective	Supports that fasting/short holds may not normalize risk
[10]	Wu et al. (2024), Can J Anaesth	Historical cohort	Adults undergoing endoscopy under anesthesia	Institutional fasting policy	Endoscopy report	Presence of gastric contents	GLP-1 RA use linked to higher odds of retained contents	Corroborates the association between GLP-1 use and retained contents
[11]	Pai et al. (2025), Anesth Analg	Prospective observational	Adults on GLP-1 RAs presenting for anesthesia	Real-world fasting/holds (varied)	Gastric ultrasound	Increased RGC	Non-trivial RGC burden despite fasting; supports PoCUS	Justifies PoCUS for individualized management
[12]	O'Brien et al. (2025), SPA Poster	Prospective pediatric cohort (abstract)	Children on GLP-1 RAs vs controls	Standard pediatric NPO	Gastric ultrasound	RGC prevalence	Pediatric GLP-1 users had greater retained contents	Pediatric signal paralleling our case
[13]	Beam and Hunter Guevara (2023), APSF Newsletter	Case reports (adult)	Adults on semaglutide under anesthesia	Guideline-compliant fasting	Clinical course	Regurgitation/aspiration events	Two cases of retained solids and regurgitation; safety alert	Illustrates severity; motivates full-stomach precautions
[14]	Ukwade et al. (2025), Front Med (Lausanne)	Retrospective cohort	1,200+ outpatient EGDs; GLP-1 users vs non-users	Standard EGD fasting; subset had same-day colon prep	Endoscopy report	Retained gastric contents	GLP-1 users had higher retained contents; colon prep protective	Aligns with bowel-prep/clear-liquid context
[15]	Kindel et al. (2025), Clin Gastroenterol Hepatol	Multisociety clinical practice guidance	Adult and pediatric populations (multidisciplinary consensus)	Advises a 24-hour liquid diet, individualized assessment	Review of existing guidelines	Risk-stratified approach; use PoCUS, use RSI, or defer procedure in high-risk patients	Reflects our patient's management (liquid diet + PoCUS + RSI)	Aligns with our management: 24-hour liquid diet + PoCUS + RSI in high-risk pediatric GLP-1 RA
[16]	Chang et al. (2024), J Clin Med	Scoping review	17 studies of adult and pediatric GLP-1 users	Variable; most followed fasting/hold guidelines	Literature review	GLP-1 users show increased gastric content/regurgitation; supports individualized management	Reinforces our case's emphasis on individualized risk assessment and shared decision-making in children on GLP-1 therapy	Backs PoCUS + individualized planning; GLP-1 users show higher gastric contents

## Conclusions

As the use of GLP-1 RAs continues to rise in the pediatric population, anesthesia clinicians must be vigilant to mitigate the risks of associated delayed gastric emptying and increased aspiration. As a result, standard fasting protocols may not be sufficient in this population. This case highlights the importance of individualized perioperative planning, including the consideration of extended fasting, gastric ultrasound assessment, and the use of rapid sequence induction when appropriate. In our case, the patient's underlying neurodevelopmental conditions may have influenced her understanding of fasting instructions, creating the possibility of an unrecognized deviation from NPO guidelines despite close caregiver supervision. There is an urgent need for updated evidence-based perioperative guidelines to ensure the safety of this growing patient population. 
